# Systematic Identification and Characterization of Long Non-Coding RNAs in the Silkworm, *Bombyx mori*

**DOI:** 10.1371/journal.pone.0147147

**Published:** 2016-01-15

**Authors:** Yuqian Wu, Tingcai Cheng, Chun Liu, Duolian Liu, Quan Zhang, Renwen Long, Ping Zhao, Qingyou Xia

**Affiliations:** 1 School of Life Sciences, Chongqing University, Chongqing 400044, China; 2 State Key Laboratory of Silkworm Genome Biology, Southwest University, Chongqing 400715, China; Institute of Plant Physiology and Ecology, CHINA

## Abstract

Long noncoding RNAs (lncRNAs) are emerging as important regulators in various biological processes. However, to date, no systematic characterization of lncRNAs has been reported in the silkworm *Bombyx mori*. In the present study, we generated eighteen RNA-seq datasets with relatively high depth. Using an in-house designed lncRNA identification pipeline, 11,810 lncRNAs were identified for 5,556 loci. Among these lncRNAs, 474 transcripts were intronic lncRNAs (ilncRNAs), 6,250 transcripts were intergenic lncRNAs (lincRNAs), and 5,086 were natural antisense lncRNAs (lncNATs). Compared with protein-coding mRNAs, silkworm lncRNAs are shorter in terms of full length but longer in terms of exon and intron length. In addition, lncRNAs exhibit a lower level of sequence conservation, more repeat sequences overlapped and higher tissue specificity than protein-coding mRNAs in the silkworm. We found that 69 lncRNA transcripts from 33 gene loci may function as miRNA precursors, and 104 lncRNA transcripts from 72 gene loci may act as competing endogenous RNAs (ceRNAs). In total, 49.47% of all gene loci (2,749/5,556) for which lncRNAs were identified showed sex-biased expression. Co-expression network analysis resulted in 19 modules, 12 of which revealed relatively high tissue specificity. The highlighted darkgoldenrod module was specifically associated with middle and posterior silk glands, and the hub lncRNAs within this module were co-expressed with proteins involved in translation, translocation, and secretory processes, suggesting that these hub lncRNAs may function as regulators of the biosynthesis, translocation, and secretion of silk proteins. This study presents the first comprehensive genome-wide analysis of silkworm lncRNAs and provides an invaluable resource for genetic, evolutionary, and genomic studies of *B*. *mori*.

## Introduction

Long non-coding RNAs (lncRNAs) have been arbitrarily defined as non-coding RNAs greater than 200 nucleotides in length. A generally used criterion for distinguishing from non-coding RNAs and protein-coding RNAs, is that the former do not encode an open reading frame (ORF) of more than 100 amino acids (aa) [1]. Similar to mRNAs, lncRNAs are subject to post-transcriptional modifications such as capping, polyadenylation, and splicing [2]. Putting protein-coding genes as reference, lncRNAs are transcribed from intronic or intergenic regions of the genome in a sense or antisense orientation. During the last decade, lncRNAs have attracted much attention due to their important roles in regulating complex biological processes. LncRNAs are capable of interacting with DNA and/or proteins to generate modular scaffolds for transcriptional gene silencing, alternative pre-mRNA splicing, direct modification of chromatin and chromosome architecture, and protein degradation [3, 4].

Unlike microRNAs, lncRNAs are generally less conserved in terms of nucleotide sequence across phylogenetically related species, making it difficult to detect lncRNAs by sequence similarity searching [5]. Next-generation sequencing technologies have emerged as powerful tools for exploring whole-genome lncRNA. A human transcriptome analysis of thousands of tumors, normal tissues, and cell lines yielded 90,013 expressed genes, of which 68% (58,648) were classified as lncRNAs [6]. In addition, more than 8,000 lncRNAs have been identified in mouse testis during postnatal testis development [7]. Although less well characterized than vertebrates and plants, to our knowledge, thousands of lncRNAs have been identified in three insect species [6, 8–17]. In the fruit fly (*Drosophila*), up to 4,000 candidate lncRNA genes were identified, resulting in a catalog of about 1,875 lncRNAs producing 3,085 transcripts [18]. Approximately 3,008 genic and 6,855 intergenic lncRNAs (lincRNAs) were identified by deep midgut transcriptome annotation [14]. In *Anopheles gambiae*, 2,949 lncRNAs have been identified in samples representing multiple life stages using deep RNA-seq technology [19]. More recently, Jayakodi identified 1,514 lincRNAs in *Apis mellifera* and 2,470 lincRNAs in *Apis cerana*, and investigated their response to viral infection [15]. Functionally, several lncRNAs have been experimentally validated as important regulators of gene regulation, dosage compensation, development, and behavior in the fruit fly. For instance, lncRNA hswω-n transcript forms perinuclear omega-speckles in nuclei in response to heat shock [20]. Two male-specific lncRNAs, *roX1* and *roX2*, present in the male-specific lethal (MSL) protein complex play pivotal roles in targeting chromosome-wide modification for dosage compensation in *Drosophila* [21]. *Yellow-achaete intergenic RNA* (*yar*) lncRNA serves as a regulator of yellow and achaete gene transcription to alter sleep regulation in the context of a normal circadian rhythm in the fruit fly [22]. The neural-specific *Drosophila* lncRNA *CRG* (CASK regulatory gene) participates in locomotion and climbing by enhancing its neighboring *CASK* expression via the recruitment of RNA polymerase II to the *CASK* promoter regions [23]. Another example of a behavior-related *Drosophila* lncRNA is *Sphinx*, whose 5'-flanking 300-bp sequence is conserved across *Drosophila* species. The *Sphinx* lncRNA is involved in regulating courtship behavior [24]. In *Apis mellifera*, only six lncRNAs have been experimentally confirmed to date, of which four (*Nb-1*, *Ks-1*, *AncR-1*, and *kakusei*) are preferentially expressed in the brain and related to behavior [25–29] and the other two (*lncov1* and *lncov2*) are expressed in the ovaries. *lncov1*, which is overexpressed in the ovaries of worker bees, is associated with transgressive ovary size [30].

The silkworm, which is a lepidopteron model insect of economic importance, has huge value for studying the fundamental mechanisms of non-coding gene regulation [31]. To date, efforts have been made to study non-coding RNA of silkworm. Genome-wide analysis has revealed a landscape of microRNAs [32, 33], snoRNA [34], and PIWI-interacting RNAs [35]. However, silkworm lncRNAs remain poorly characterized. To the best of our knowledge, only one silkworm lncRNA (*Fben-1*), which is preferentially expressed in the female brain, has been reported to date [36]. The systematic screening of potential lncRNAs in the silkworm genome has not yet been reported. In this study, we performed deep transcriptome sequencing of 18 tissue samples collected from fifth instar silkworm larvae. By combining our data with 2 additional public silkworm RNA-seq datasets (which represented 3 tissue samples), we systematically identified lncRNAs at the whole-genome level. Our results indicate that a large number of silkworm lncRNAs show relatively low expression levels, high spatial specificity, and low levels of sequence conservation compared with silkworm protein-coding mRNAs. These lncRNAs may serve as miRNA precursors or ceRNAs, and are suspected to be involved in miRNA regulatory pathways. In addition, our results reveal that a proportion of lncRNAs in the silk gland gene co-expression network core may participate in the biosynthesis, translocation, and secretion of silk proteins.

## Materials and Methods

### Silkworm rearing and tissue collection

The silkworm strain *Dazao* were obtained from the Silkworm Gene Bank of Southwest University, Chongqing, China. All larvae were reared at 25°C, 60% relative humidity, with a 16:8 h light-dark regimen, and fed with mulberry leaves. Sexed tissues including the anterior silk gland (ASG), the anterior section of middle silk gland (AMSG), the middle section of middle silk gland (MMSG), the posterior section of middle silk gland (PMSG), the posterior silk gland (PSG), gonad (testis/ovary), fat body, Malpighian tubule (MpT), and brain were dissected from day 3 fifth instar male and female larvae, respectively. All samples were frozen immediately in liquid nitrogen and stored at -80°C until use.

### RNA extraction, library construction, and sequencing

Total RNA was extracted from silkworm tissues using the TRIzol reagent (Invitrogen) and further purified with the RNeasy kit (Qiagen). The integrity and quality of RNA were assessed using the Agilent 2100 Bioanalyzer (Agilent technologies). For non-strand-specific libraries, mRNAs were selected using oligo(dT) magnetic beads (Invitrogen), fragmented, and used to synthesize cDNA according to the TruSeq RNA Sample Preparation v2 Guide (Illumina). For strand-specific libraries, ribosomal RNA was depleted using Ribo-Zero rRNA removal beads. Then, the total RNA was purified and fragmented in fragmentation buffer. Next, the strand-specific sequencing libraries were constructed using TruSeq Stranded Total RNA Sample Preparation kits (Illumina, San Diego, CA). Libraries were sequenced on the Hiseq2000 system (Illumina, San Diego, CA). All RNA sequencing data produced in present study have been deposited in NCBI Short Read Archive (http://www.ncbi.nlm.nih.gov/sra/) and can be accessed under the SRA accession number: PRJNA284192.

### Public available RNA-seq data

RNA-seq data from early-sexed embryonic stages of silkworm were obtained from a previously published study [37] and downloaded from the NCBI SRA website under the accession number DRA001104. RNA-seq data for the integument (GenBank accession numbers PRJNA215013 and PRJNA238971), previously reported by our group, were also included in this study [38].

### Mapping of RNA-seq reads

The quality of raw reads was evaluated using FastQC [39]. Raw reads were filtered and trimmed using Trimmomatic 0.32 (parameters: ILLUMINACLIP: TruSeq3-PE.fa:2: 30:10; HEADCROP:10; TRAILING:3; SLIDINGWINDOW:4:20; MINLEN:75) [40]. Remaining reads were mapped against silkworm rRNA, tRNA, and mtDNA sequences collected in-house, using bowtie2 (version 2.2.3, parameters:–N 1;–L 20;–k 20), and matching reads were discarded [41]. The remaining high-quality clean reads were mapped to the silkworm genome (SilkDB 2.0 release) [42] using the spliced read aligner TopHat (version 2.09) [43]. In order to maximize the usage of splice junction information derived from all tissues, the previously described two rounds of TopHat mapping strategy was adopted [44]. In brief, reads from each samples were mapped with TopHat using the default parameters except ‘min-anchor = 5’ and ‘min-isoform-fraction = 0’. All splice junctions detected by initial mapping were pooled and used as raw junctions for the second round of mapping, with the following parameters: ‘raw-juncs’, ‘no-novel-juncs’. In order to facilitate transcript assembly and quantification, all mapped reads from the same tissue were merged into a single BAM file.

### Transcriptome assembly

The transcriptome of each tissue was assembled from the TopHat mapped reads separately by Cufflinks [45], Scripture [46], and StringTie [47]. Cufflinks (version 2.02) was run with default parameters (and ‘min-frags-per-transfrag = 0’), Scripture (VPaperR 3) was run with default parameters (and omission of the ‘-pairedEnd’ option), StringTie (version 1.0.1) was run with the parameters (-f 0.01 -a 10 -j 1 -c 0.01), which from the slightly alter default parameters. The transcripts which was supported by at least two assembly programs or occurred in at least two tissues, was extracted as stringent transcripts. Stringent transcripts were merged into a unique transcript set using Cuffmerge. Then, the read coverage and fragments per kilobase of transcript per million mapped reads (FPKM) values for the 21 tissue types were estimated using Cufflinks.

### LncRNA identification pipeline

**Fig 1:**
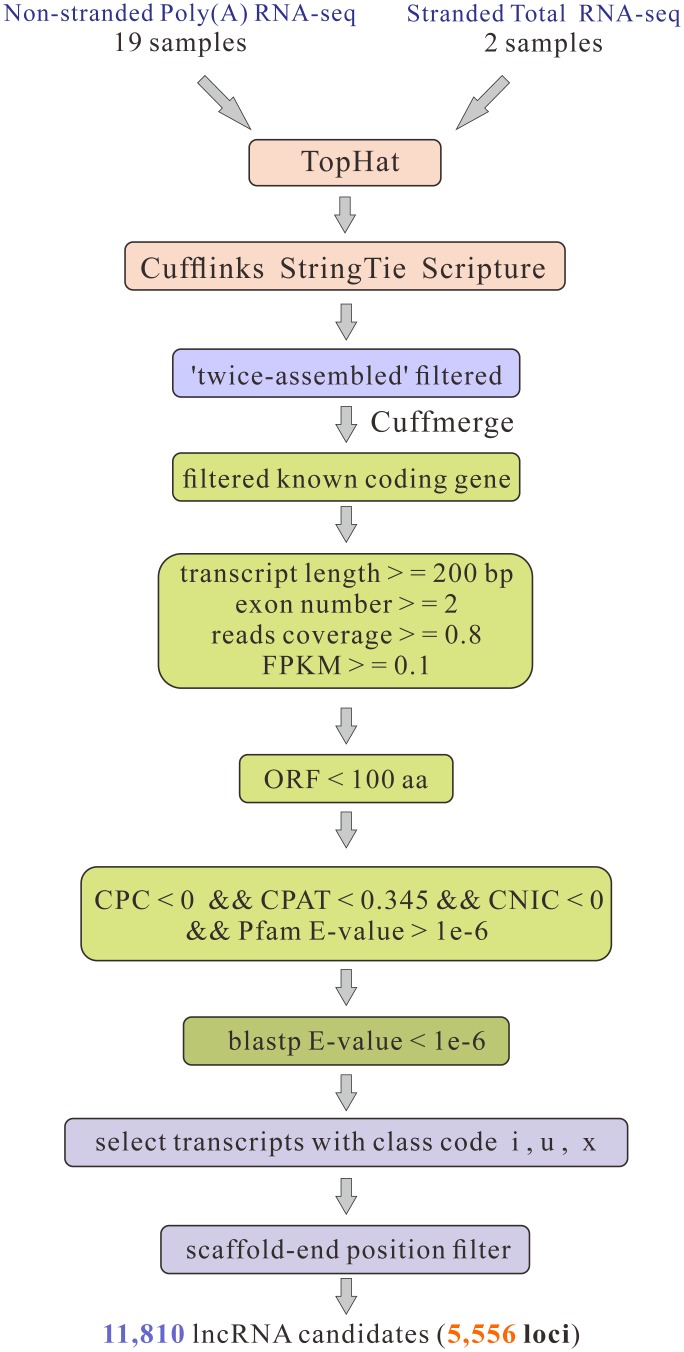
Integrative computational pipeline for the systematic identification of lncRNAs in silkworm. FPKM, Fragments per kilobase of transcript per million mapped reads; ORF, open reading frame; CPC, coding potential calculator; CPAT, RNA coding potential assessment tool; CNIC, coding non-coding index.

We developed an analysis pipeline to identify bona fide lncRNAs from the newly generated silkworm transcriptome (Fig 1). (1) Transcripts that overlapped with any protein-coding exon in the sense orientation were removed; (2) transcripts with < 200 bp, single-exon, read coverage < 0.8, and FPKM < 0.1 were eliminated; (3) transcripts with predicted large ORFs (> 100 aa) were filtered out; (4) transcripts with predicted protein-coding potential were removed (protein-coding potential criteria: CPC score > 0, CPAT score > 0.345, and CNIC score > 0) [48–50]; (5) transcripts with similarity to known protein sequences in the Swiss-Prot database (E-value < 1e-6) [51] and known protein-coding domains in the Pfam (AB) database (E-value < 1e-6) [52] were discarded; (6) transcripts within the < 2k scaffold-end range were excluded; (7) finally, transcripts with class code ‘i’,‘u’,’x’ subsets were retained as bona fide silkworm lncRNAs.

### Analysis of sequence conservation of silkworm lncRNAs

The sequence conservation of silkworm lncRNAs was evaluated based on sequence similarity search, using the previously described method [2]. The genomes of *Caenorhabditis elegans*, *Acyrthosiphon pisum*, *A*. *mellifera*, *A*. *gambiae*, *Drosophila melanogaster*, *Tribolium castaneum*, *Heliconius melpomene*, *Danaus plexippus*, *Melitaea cinxia*, *Mnemiopsis leidyi*, *Solenopsis invicta*, and *Tetranychus urticae* were downloaded from ENSEMBL database [53]. The genomes of *Plutella xylostella* and *Manduca sexta* were obtained from the Diamondback moth genome database (http://iae.fafu.edu.cn/DBM/) and Agripest Base (http://agripestbase.org/manduca/), respectively. The genomes of *Papilio polytes* and *Papilio xuthus* were obtained from PapilioBase (http://papilio.nig.ac.jp/index.php). The genome of *Papilio glaucus* was downloaded from the official website for the tiger swallowtail genome (http://prodata.swmed.edu/LepDB/). The lncRNA sequences were searched against these 18 genomes using BLASTN (with E-value < 1e-10); the best hit for each query and for each genome was retained, and a matrix of lncRNA/homolog pairwise similarity was obtained. The similarity matrix was visualized using the pheatmap R package [54].

### Tissue specificity score

The tissue-specific score (JS score) has been previously defined by Cabili et al. [44]. In the current study, we calculated the tissue-specific score for each transcript using the csSpecificity() function provided by the CummeRbund R package [55].

### LncRNAs as precursors of miRNAs

In order to identify lncRNAs as precursors of miRNAs, we intersected the GFF (Generic Feature Format) file containing silkworm lncRNA genomic positions with the GFF file containing mature miRNA sequences downloaded from miRBase (Release 21) [56]. LncRNA loci that overlapped with miRNA loci on the same strand were considered as the precursors of these miRNAs.

### Prediction of competing endogenous RNAs (ceRNAs)

CeRNAs may be identified by traditional miRNA target prediction methods [57–59]. In the present study, we inferred the conserved regions of silkworm lncRNAs that may harbor miRNA response elements (MREs) for ceRNA network. MREs in the conserved regions of lncRNAs were predicted using miRanda [60], PITA [61], and RNAhybrid [60].

### Weighted gene co-expression network analysis

All transcripts, including protein-coding mRNAs and lncRNAs, expressed in at least 2 samples were used for constructing the weighted gene co-expression network (WGCNA). WGCNA construction and module detection were performed using the “WGCNA” R package v. 1.4.6 [62]. The overall procedure involved the generation of a Pearson correlation matrix between all transcript pairs, followed by the transformation of this correlation matrix into an adjacency matrix with a soft-thresholding power of 9, using an adjacency function that implement in WGCNA package. Then, the adjacency matrix was transformed into a topological matrix (TOM). Primary modules were identified via linkage hierarchical clustering with the topological overlap dissimilarity matrix (1-TOM), those with high correlation (module eigengene correlation > 0.70) were merged, and the module membership (kME) of each gene was calculated. Cytoscape v.3.2.1 software was used for network visualization [63].

### Gene ontology and pathway analysis

Gene Ontology (GO) enrichment analysis of each module was performed using GOseq [64]. KEGG metabolic pathway enrichment analysis was carried out using KOBAS 2.0 [65], with the *Bombyx mori* database as background. All data were statistically analyzed using the hypergeometric test and Benjamini-Hochberg FDR (false discovery rate) correction, and only GO terms or KEGG pathways with corrected *p*-values of less than 0.05 were considered enriched.

### Identification of sex-biased transcripts

In order to identify sex-biased transcripts among the ten sex-sampled tissues, the raw read counts of each transcript were re-estimated using RSEM [66]. Fold change (FC) and FDR between females and males were calculated for each tissue type using DEGseq [67]. For a given tissue, transcripts with a |log2FC| > 1 and FDR < 0.05 were considered sex-biased.

## Results and Discussion

### Genome-wide identification of lncRNAs in silkworm

In order to systematically identify lncRNAs in the silkworm genome, we sequenced 18 libraries. Totally, 2.15 billion raw reads were generated and 1.71 billion clean reads were retained after stringent filtering (S1 Table). In addition, two public datasets from silkworm embryo and integument were also included in this study (S1 Table). In order to obtain a comprehensive silkworm transcriptome, reads from each tissue were assembled using the three most widely used assemblers (Cufflinks, Scripture, and StringTie). A total of 6,524,370 transcripts were generated, of which 3,511,465 transcripts were assembled at least twice (i.e. these transcript were assembled by at least two assemblers, or assembled in at least two tissues). We defined these ‘twice-assembled’ transcripts as stringent transcripts. The stringent transcripts were merged into a unique transcript set, composed of 29,416 gene loci and 553,658 transcripts, using Cuffmerge.

An lncRNA identification pipeline was developed as shown in Fig 1. Briefly, we filtered out transcripts that overlapped with coding gene exons in the sense orientation, retaining 55,739 transcripts for 17,553 gene loci. In order to obtain long, oriented, and expressed transcripts, we filtered out the transcripts shorter than 200 bp, those that possessed only a single exon, as well as transcripts with single base read coverage < 0.8 and FPKM < 0.1. In addition, transcripts with ORFs > 100 aa were discarded. Then, the protein coding potential of each transcript was accessed by CPC, CPAT, and CNIC, respectively. Transcripts with CPC score > 0, CPAT score > 0.345, or CNIC score > 0 were excluded. The remaining transcripts were subjected to protein domain filtering using HMMER (version 3.0) against known protein domains documented in the Pfam (version 27.0) database, in order to evaluate whether they contained a known protein-coding domain. In order to rule out incompletely assembled transcripts due to the effects of scaffold-end boundaries, transcripts within < 2k scaffold-end range were excluded. Finally, only transcripts with class codes ‘i’, ‘u’, ‘x’, representing intronic, lncRNAs (ilncRNAs), lincRNAs, and natural antisense lncRNAs (lncNATs), respectively, were retained. This resulted in a final set of 11,810 silkworm lncRNA transcripts from 5556 loci, of which 474 were ilncRNAs, 6,250 were lincRNAs, and 5,086 were lncNATs. The genomic coordinates of the identified lncRNA transcripts (GTF format) are provided in S2 Table.

In the present study, we report the generation of a relatively robust list of silkworm lncRNAs. As most of the RNA-seq libraries in this study were prepared from day 3 fifth instar larvae by the non-strand-specific poly(A) selection method, it was expected that use of a broad variety of tissues would result in identification of a larger number of lncRNAs. As most of the RNA-seq libraries were non strand-specific and poly(A)-selected, several limitations should be addressed: first, a large proportion of non-poly(A) silkworm lncRNAs could not be detected; second, single-exon transcripts were excluded due to lack of strand information; third, the number of ilncRNAs and lncNATs were underestimated. The “twice-assembled” filter strategy, that has been adopted in several studies, was utilized to prevent mis-assembly of transcripts [10, 68]; however, some bonafide transcripts may have been lost. The combination of protein-coding potential filtering and protein-domain filtering steps, has been shown efficiently reduce false negative and false positive rate for distinguishing non-coding transcripts from protein-coding transcripts [10, 44]. In addition, some transcripts assembled in the scaffold-end region may have been incompletely assembled. Scaffold-end boundary effects should be avoided for unfinished genome with a large number of scaffolds, e.g. the silkworm genome. In summary, our approach, which was comparable to previously reported methods [8–12, 68, 69], resulted in the reliable identification of lncRNAs; however, a proportion of bona fide lncRNAs may have been filtered out.

### The genomic features of silkworm lncRNAs

**Fig 2:**
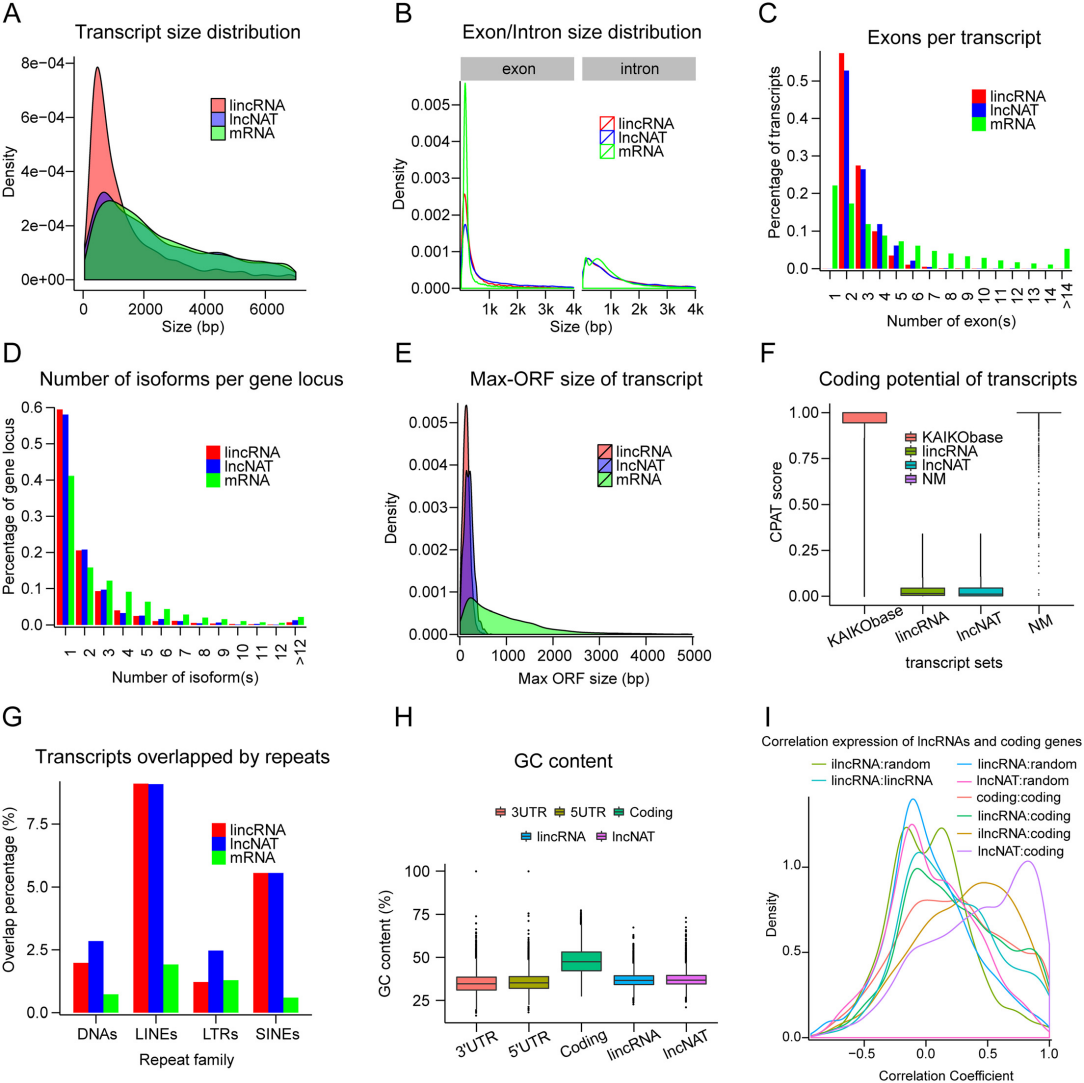
Features of silkworm lncRNAs. (A) Transcript size distribution for lincRNAs, lncNATs, and mRNAs. (B) Exon (left) and intron (right) size distributions for lincRNA, lncNATs, and mRNAs (C) Number of exons per transcript for all lincRNAs, lncNATs, and mRNAs. (D) Distribution of the number of isoforms for each lincRNA, lncNAT, and mRNA gene locus. (E) Maximum ORF size distribution for lincRNAs, lncNATs, and mRNAs. (F) CPAT score distribution for KAIKObase gene model, ‘NM_’ reference sequence of silkworm, lincRNAs, and lncNATs. (G) Proportion of lincRNAs, lncNATs, and mRNAs transcripts covered by main repeat classes annotated by RepeatMasker. (H) GC content of lincRNAs, lncNATs, and mRNAs. (I) Pearson correlation coefficient distribution for neighboring transcript pairs from different datasets. Coding, protein-coding mRNAs; Random, random shuffle of mRNA positions.

In order to characterize their genomic features, potential silkworm lncRNAs were compared with known protein-coding mRNAs. Overall, silkworm lncRNAs (median of length 1,459 bp for lncRNAs; 955 bp for lincRNAs) were found to be significantly shorter than protein-coding mRNAs (2,741 bp for mRNAs, Kolmogorov-Smirnov test (KS-test) *p*-value < 2.2e-16), whereas, lncNATs (median of length 2,602 bp) were similar in length to protein-coding transcripts (KS-test *p*-value = 0.03179) (Fig 2A). In contrast to the overall length of lncRNAs, their exon lengths (median length of 285 bp for lncRNAs, 239 bp for lincRNAs, and 405 bp for lncNATs) are significantly longer than those of protein-coding mRNAs (median length of 168 bp; KS test *p*-value < 2.2e-16). A similar pattern was observed for introns (Fig 2B); silkworm lncRNAs have fewer exons than mRNAs (2.73 vs. 5.17 on average; 2.66 for lincRNAs, 2 for lncNATs; KS test *p*-value < 2.2e-16) (Fig 2C). This finding may explain the longer exon length and shorted overall length of lncRNAs relative to mRNAs. LncRNA loci possess fewer transcript isoforms than protein-coding mRNA loci (2.03 vs. 3.18 on average per gene locus, 1.97 for lincRNA, 2.11 for lncRNAs, KS test *p*-value < 2.2e-16) (Fig 2D), suggesting that lncRNAs are less complex than protein-coding mRNAs. The median sizes of the max-ORF of lncRNAs (138 bp for lincRNAs and 189 bp for lncNATs) are significantly shorter than those of mRNAs (732 bp for mRNAs, KS test *p*-value < 2.2e-16) (Fig 2E). Analysis using the Wilcoxon rank sum test show that silkworm lncRNAs have lower protein-coding potential than well-annotated KAIKObase gene models and NM annotations downloaded from NCBI (Fig 2F). Similar to mammalian lncRNAs, silkworm lncRNAs contain more repeat sequences than mRNAs (18.7% vs. 4.54%) (Fig 2G). The predominant repeat sequences within lncRNAs are LINEs (7.9%) and SINEs (6.5%). For the four main classes of repeats (LINE, SINE, DNA, and LTR), except LTR, both lincRNAs and lncNATs show a greater preference overlapped with repeat elements than mRNA (Fig 2G). Interestingly, the GC content in silkworm lncRNAs is lower than in coding sequences (CDS) but slightly higher than in untranslated regions (UTRs) (Fig 2H). The expression profiles of silkworm lincRNAs did not show stronger correlation with the adjacent protein-coding genes. However, like the nearby coding gene pairs, lincRNAs tend to correlate with their nearest protein-coding neighbors compared with randomly selected counterparts (Fig 2I). In about 80% of lncNATs, only a short fraction of the length (less than 35% of the sequence) is overlapped by protein-coding exons (S1D Fig), whereas lncNATs show relatively high correlation with antisense protein-coding genes (Fig 2I).

Silkworm lncRNAs exhibit numerous features that are distinct from those of coding mRNAs; however, the vast majority of lncRNAs are spliced by canonical splices sites (GT/AG), and no differences in splicing signal usage are found compared with protein-coding mRNAs (S2B Fig). In addition, the distribution of lncRNAs in silkworm chromosomes was examined. Silkworm lncRNAs were unevenly distributed across the 28 silkworm chromosomes (Chi-Square Goodness of Fit Test, *p*-value < 2.2e-16) (S1A Fig, S3 Table). Intriguingly, chromosome Z contained the largest number of lncRNAs with the highest gene density (722 transcripts, 304 gene loci, 14.9 genes/Mb), whereas the smallest chromosome (chromosome 2) presented the least number and lowest density of lncRNAs (108 transcripts, 64 gene loci, 8.1 genes/Mb).

The levels of conservation and polymorphism of lncRNAs were also investigated, as shown in Fig S2C. We found that 58.3% lncRNAs (6,885) were specific to silkworm, in contrast with 17.9% of coding mRNAs (2,990). Homologous fragment sequences of 41.44% lncRNAs (4,894/ 11,810) can be found in other Lepidoptera species. In contrast, only 12.01% of lncRNAs (1,419/11,810) were found to be conserved when comparing with the slightly more distantly related species *A*. *pisum*, and only 1.74% of lncRNAs (206/11,810) were found to be conserved when comparing with even more distantly related species. For coding mRNAs, 63.14% (10,521/16,664) were conserved among the representative eighteen species. LincRNAs possessed a larger number of polymorphism sites than lncNATs and ilncRNAs (Fig S2E). These results suggest that silkworm lncRNAs are highly species-specific and conserved to a small extent among Lepidopterans. In addition, silkworm lncRNAs are considered to have undergone more rapid evolution than protein-coding mRNAs do.

Collectively, silkworm lncRNAs share similar patterns with those of other species such as flies, humans, and zebrafish. In particular, silkworm lncRNAs possess short exons, long introns, low levels of conservation, low GC content, and a large degree of overlap of repeat sequences. Additionally, silkworm lncRNAs are slightly related to their closest protein-coding neighbors. In addition, we found that the transcript lengths of lncNATs were similar to those of mRNAs compared with those of lincRNAs, although both types of non-coding RNAs (lncNATs and lincRNAs) shared several common features.

However, due to most of our RNA-seq libraries were non-strand specific, single-exon lncRNAs were excluded from this study, even though the silkworm, like other species, is considered to possess a large proportion of single-exon lncRNAs. It must be noted that the transcript lengths and exon numbers per transcript of lncRNAs may have been overestimated, and that the number of lncNATs and exon sizes of lncRNAs may have been underestimated in the present study. Previous studies have shown that lncRNAs exhibit more positional conservation than sequence conservation across species, and revealed that the positional conserved lncRNAs have important biological functions [70, 71]. The present study focused on the analysis of sequence conservation, rather than positional conservation, in silkworm lncRNAs, which is not suitable for inferring the functions of the conserved lncRNAs. Therefore, in order to elaborate the functional role of conserved lncRNAs, the further studies based on phylogenomic approach are needed.

### Silkworm lncRNAs are more tissue-specific than mRNAs

**Fig 3:**
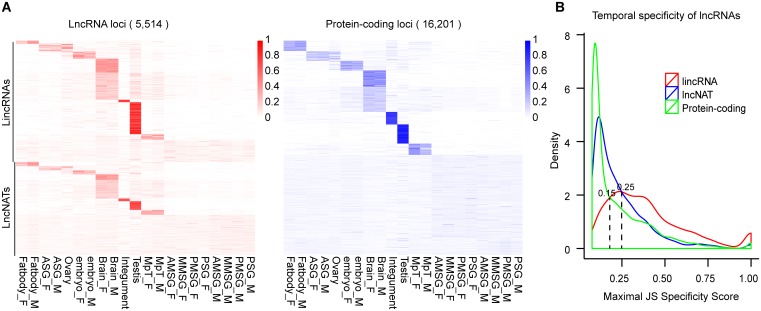
Tissue specificity of lncRNAs and protein-coding genes. (A) Heatmaps of 5,514 lncRNA loci (red; left) and 16,201 protein-coding loci (blue; right) based on normalized expression values (the sum of expression values across all tissues per locus is set to one, using the method described by Cabili, M. N., et al [44]). (B) The distribution of maximal tissue specificity scores for each transcript across 21 tissues.

Based on the FPKM values of genes estimated using Cufflinks, tissue-specific lncRNAs were investigated in the 21 silkworm tissues by determining the tissue specificity score, also termed the JS (Jensen-Shannon) score [44]. JS scores range from zero for genes expressed ubiquitously in all tissues, to one for genes expressed in a single tissue (Fig 3 and S2 Fig). Using JS score = 0.25 as a cutoff, the majority of lincRNAs (73.5%) were found to be tissue-specific, compared with 34.6% mRNAs. In contrast, 39.1% of lncNATs were tissue-specific (Fig 3A). Moreover, more than a third of all lincRNAs were specific to the testis and approximately one fifth were specifically expressed in the brain. These findings are consistent with previous reports [5, 44]. KS test revealed that lincRNAs show much higher tissue specificity than lncNATs (*p*-value < 2.2e-16). Furthermore, both lincRNAs and lncNATs show greater tissue specificity than mRNAs (*p*-value = 2.23e-13) (Fig 3B).

The expression levels of silkworm lncRNAs (both lincRNAs and lncNATs) were significantly lower than those of protein-coding genes in the 21 tissues (KS test *p*-value < 2.2e-16 for lincRNAs vs. mRNAs, *p*-value < 2.2e-16 for lncNATs vs. mRNAs), although lncRNAs show slightly higher levels of expression in the brain and testis than in other tissues (S2A Fig). The median maximal expression levels of lincRNAs and lncNATs are ~29-fold and ~8-fold lower than those of mRNAs (median maximal FPKM is 1.0 for lincRNAs, 3.1 for lncNATs, 1.6 for lncRNA, and 24.9 for mRNAs) (S2B Fig). Although lncRNAs are expressed at relatively lower levels than protein-coding genes, their high tissue specificity suggests that they may perform *ad hoc* biological functions in specific tissues, rather than simply contributing to transcriptional noise.

### Classification of silkworm lncRNAs as miRNA precursors and potential competing endogenous RNAs

**Fig 4:**
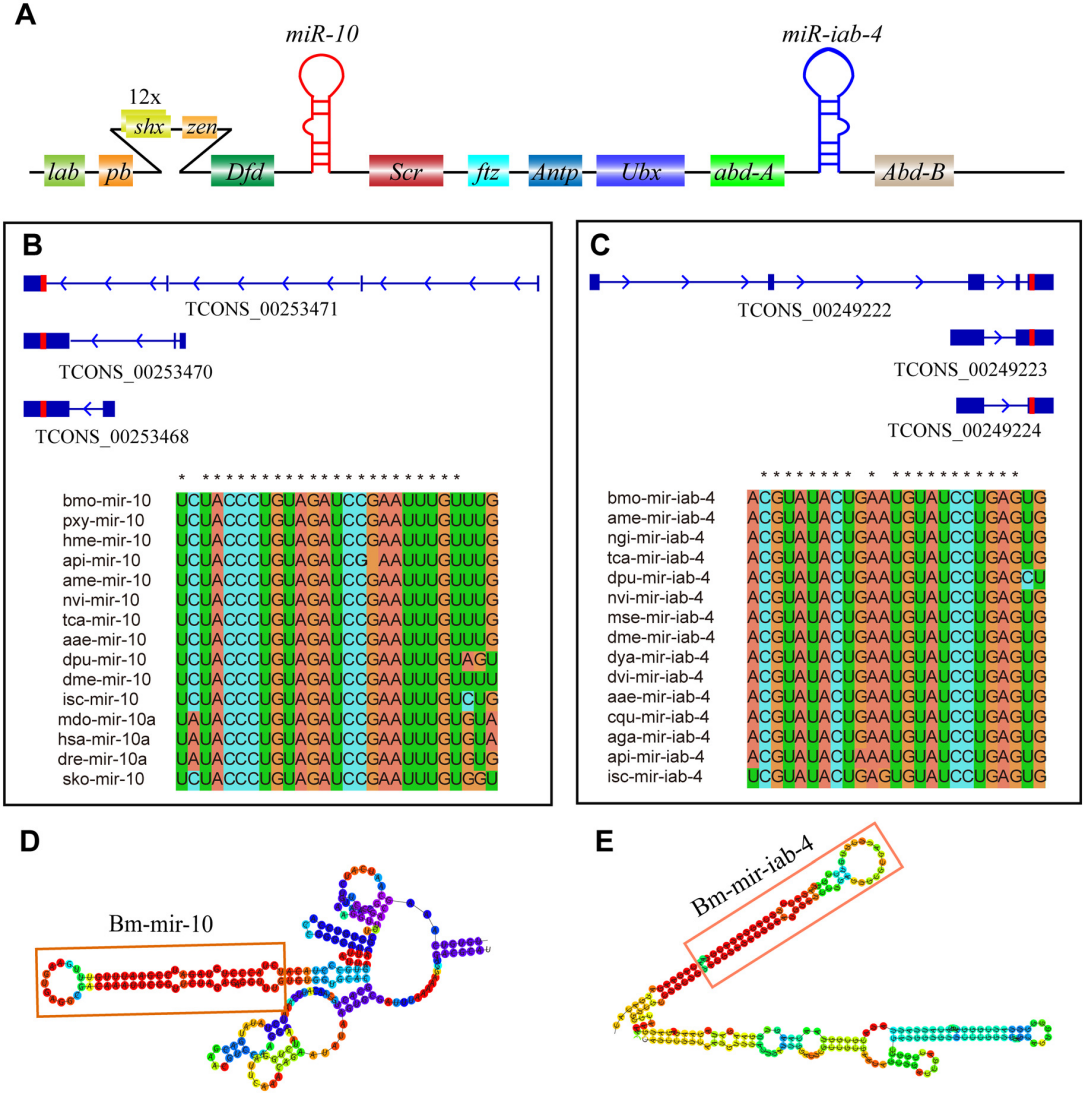
Potential miRNA precursors in the silkworm *Hox* cluster. (A) Schematic representation of the silkworm Hox cluster with potential miRNA precursors for miR-iab-4 (blue) and miR-10 (red). (B) Schematic of bmo-mir-10 precursors and alignment of bmo-miR-10 orthologues from selected bilaterians. Boxed regions indicate miR-10 primary sequences. Asterisk indicates sequences that are highly conserved with those of silkworm. (C) Schematic of bmo-mir-iab-4 precursors and alignment of bmo-mir-iab-4 orthologues from selected bilaterians. Boxed regions indicate bmo-mir-iab-4 primary sequences. Asterisk indicates sequences that are highly conserved with those of silkworm. (D) Secondary structures of bmo-mir-10 represent precursor (TCONS_). (E) Secondary structures of bmo-mir-iab-4 represent precursor (TCONS_). All secondary structures were predicted using RNAfold.

Certain lncRNAs may function as precursor molecules that are processed into smaller regulatory RNAs such as miRNAs [9, 10, 72]. In order to determine whether silkworm lncRNAs are actually precursors of miRNAs, we compared their genomic coordinates with corresponding genomic locations on the same strand of miRNAs downloaded from miRBase (Release 21). In all, 69 lncRNAs from 33 gene loci, were identified as known precursors and found to be distributed among 25 silkworm miRNA families (S4 Table). For example, tow gene loci XLOC_010603 and XLOC_010759 residing in the *Hox* gene cluster were determined to be precursors of miR-iab-4 and miR-10, respectively (Fig 4). miR-iab-4 is analogous to miR-196 in vertebrate Hox clusters. In vivo experiments in *Drosophila* showed miR-iab-4-5p directly inhibits *Ubx* activity and regulates ectopic expression of mir-iab-4-5p, resulting in the transformation of halters into wings [73]. mirR-10 was predicted to target the *Scr* gene. The 3’UTR of *Scr* genes has been conserved over hundreds of millions of years of evolution, suggesting that this region is likely the functional target site for miR-10 [74]. The transcriptional levels of bmo-miR-10b-3p/5p were significantly increased during metamorphosis [75]. Moreover, the transcript TCONS_00253471 of the XLOC_010759 loci was highly expressed in embryos and body walls. Taken together, these data suggest that the XLOC_010603 and XLOC_010759 transcripts overlapping with miR-10 and miR-iab-4 may function as miRNA precursors, playing an important role in the regulation of *Hox* gene expression.

LncRNAs have undergone rapid sequence evolution; however, some lncRNAs still possess short functional elements that have remained conserved in different species [71]. LncRNAs may bind miRNAs as competing endogenous RNAs (ceRNAs), thereby functioning as miRNA sponges [76]. The lncRNA-miRNA interaction can be examined using traditional miRNA target prediction methods [57–59]. In the current study, we inferred the conservation elements region of silkworm lncRNAs that may harbor miRNA response elements (MREs) for the ceRNA network. In total, 104 lncRNAs from 72 gene loci were predicted as ‘decoys’ for 101 known miRNA families (S5 Table). For example, the transcripts TCONS_00111202 and TCONS_00111199 from the XLOC_004695 gene locus harbor the bmo-miR-184-3p, bmo-miR-3378-5p, and bmo-miR-745-5p response elements (S3A Fig). MiR-184, a single-copy gene that is evolutionarily conserved from insects to primates, is expressed ubiquitously in *Drosophila* embryos, larvae, and adults, and shows dynamic expression pattern in the central nervous system during embryonic development [77]. Loss of function of miRNA-184 results in reduced motility in adult *Drosophila* and complete loss of egg production in the female [78, 79]. More recently, a study found that miR-184, which plays an important role in energy homeostasis, was negatively regulated upon administration of a sucrose-rich diet [80]. TCONS_00111202, expressed ubiquitously in the 21 silkworm tissues studied, exhibits slightly higher expression levels in the ASG, fat body, and embryo compared with other tissues (S3B Fig). The miRNA response elements in TCONS_00111202 are conserved from *B*. *mori* to *T*. *castaneum*, implying that TCONS_00111202 may function as a ceRNA, with important roles in the silkworm. These results reveal that lncNAs may function as miRNA precursors or ceRNAs, and play important roles in numerous regulatory pathways.

### Sex-biased expression of silkworm lncRNAs

**Fig 5:**
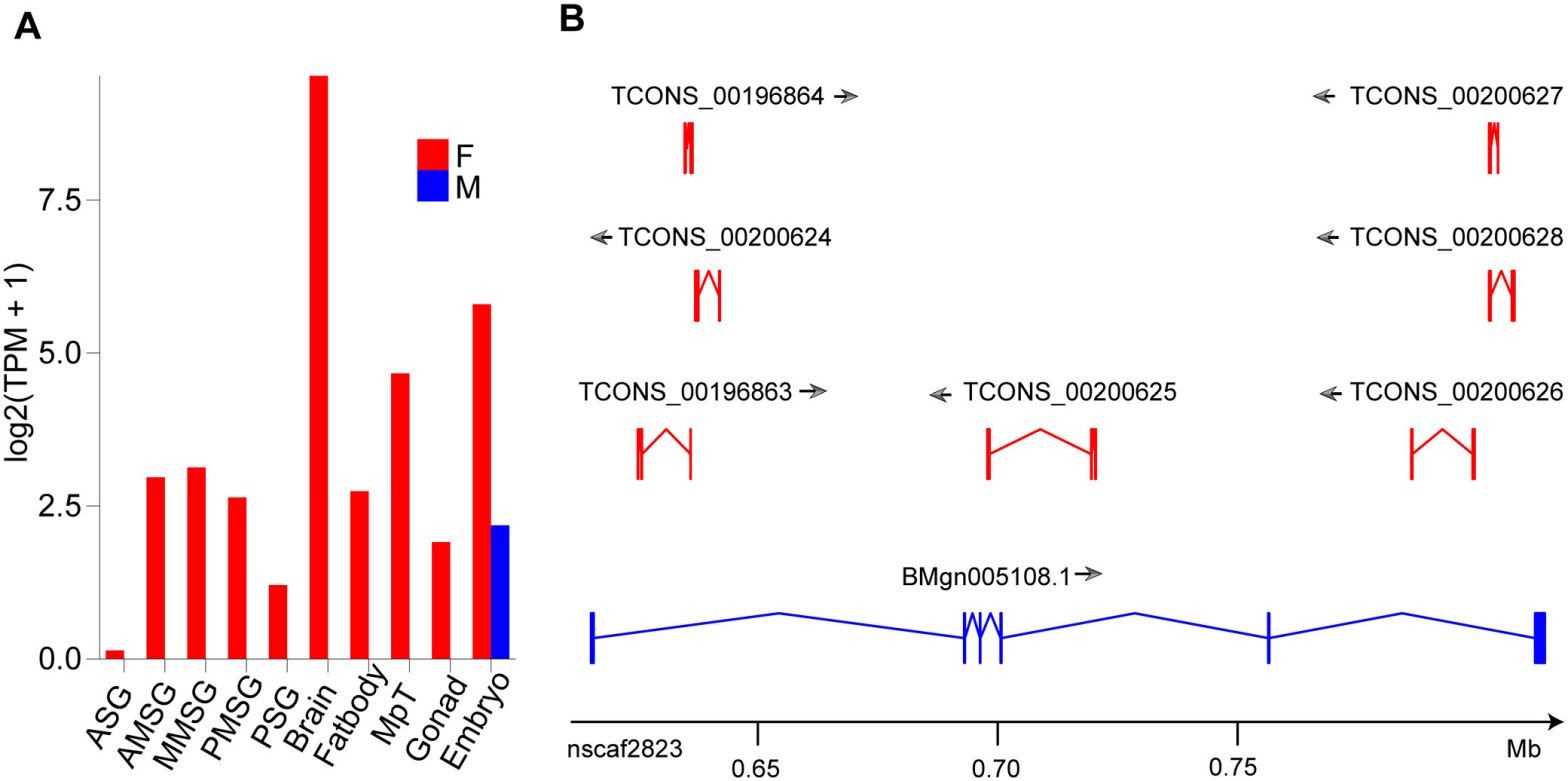
Quality of sex-sampled and sex-biased lncRNAs at the Bmdsx gene locus. (A) Expression pattern of the W chromosome-specific gene, fem. (B) Sex-biased lncRNAs at the Bmdsx gene locus.

The examination of sex-biased expression of silkworm coding genes, using microarray technology, reveals that male-biased genes are enriched on the Z chromosomes [81]. It is of great interest to investigate the sex-biased expression of silkworm lncRNAs. Firstly, in order to confirm the samples as sex-specific, the W chromosome-specific gene (*fem*) was adopted as a marker gene, as the male library may be derived from incorrectly sexed embryos or RNA produced by polar bodies [37], whereas the read counts of the *fem* gene from the male library are far fewer than those of the female library (Fig 5A). Therefore, it was ensured that the embryo samples were suitable for sex-biased analysis, whereas the integument sample yielded only one mix-sex sampled library [38]. Finally, all 20 samples were correctly sex-sampled and retained for further analysis. According to the criteria (|log2FC| > 1 and FDR < 0.05), significantly more genes were found to be upregulated in male (male-biased expressed) gonads, Malpighian tubes, brains, and PSG, whereas the reverse was observed for PMSG, AMSG and ASG (S6 Table). A similar pattern was observed for the protein-coding genes (S6 Table). Notably, lncRNAs with female-biased expression were vastly outnumbered by those with male-biased expression in the gonad (female vs. male: 774 vs. 3,176). Among these male-biased lncRNAs, 1,772 transcripts were specifically expressed in the testis and found to be enriched on Chromosome Z, 13, and 22, suggesting that the male-biased lncRNAs contribute to spermatogenesis and other male-specific biological processes.

Among the 5,556 lncRNA gene loci, 49.47% (2,749) showed sex-biased expression. In detail, 1,029 single-isoform gene loci (32.33%) showed sex-biased expression, whereas 1,720 multi-isoform gene loci, with at least one isoform, exhibited sex-biased expression. In this study, we defined a sex-biased ratio for multi-isoform gene loci in order to represent the rate of sex-biased isoforms. Gene loci with a sex-biased ratio of over 0.75 were considered sex-biased. Applying this sex-biased ratio criterion, the numbers of sex-biased gene loci were found to be 1,029 and 523 for single- and multi-isoform gene loci, respectively. Taking gene locus XLOC_012091 as an example, 14 isoforms were annotated in this study, of which 11 isoforms showed sex-biased expression and were antisense with respect to *yellow-d* (KAIKOBASE ID: BMgn007254) (S4A Fig). The *yellow*-like gene, which has only been identified in insect and bacterial species, has been reported to be involved in the melanin biosynthetic pathway and associated with movement and mating behavior in *Drosophila* [82–84]. Ten isoforms of XLOC_012091, with the exception of TCONS_00285772, TCONS_00285767, TCONS_00285768, and TCONS_00285757 exhibited testis-specific expression (S4B Fig), suggesting that the XLOC_012091 gene loci, which shows a strong male-biased expression signal, may be involved in silkworm mating behavior.

LncRNAs that were nearing, or intersecting with, primary sex determination pathway genes (*Fem*, *Masc*, *Imp*, *Psi*, *Dsx*) were searched, and two lncRNA isoforms located in the *Psi* intron region, six lncRNA isoforms in the *Dsx* intron region, and one isoform antisense to Dsx were found. Notably, TCONS_00200625 isoform, an antisense transcript, was found to overlap with the 4th exon of Dsx, suggesting that TCONS_00200625 may interfere with sex-specific splicing in Dsx (Fig 5).

In summary, numerous silkworm lncRNAs showed sex-differential expression, with some gene loci displaying very high sex-biased ratios and sex-limited expression. A few lncRNAs were identified to play an important role in sex determination pathways. Although more evidences are needed to prove these findings, our results demonstrate sex-biased expression in silkworm lncRNAs and provide supplement account for sexual dimorphism in the silkworm.

### Functional annotation of silkworm lncRNAs

**Fig 6:**
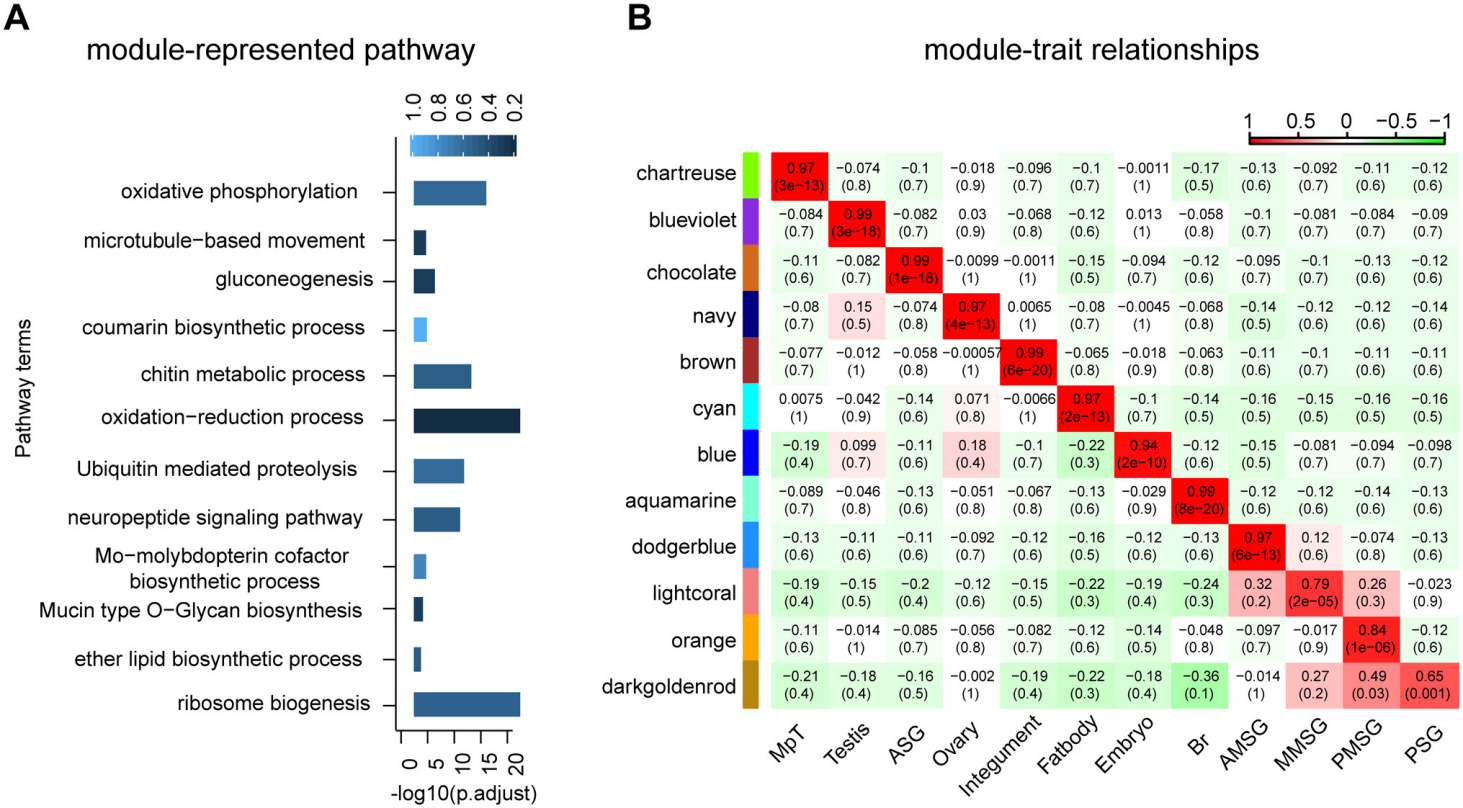
Functional enrichment of protein-coding genes in network modules and module-tissue correlation, and the corresponding *p*-values. (A) Functional enrichment of protein-coding genes in network modules. For each module, representative enrichment GO terms are shown, with bar plot of -log10 (p.adjust). Light to dark blue represent increasing enrichment factors (from 0 to 1). (B) Module-tissue correlations and corresponding *p*-values. Boxes contain Pearson correlation coefficients and their associated *p*-values. Positive correlation (red) indicates that the module is positively correlated with the specific tissue, whereas negative correlation (green) indicates the reverse. MpT, Malpighian tubule; ASG, anterior silk gland; AMSG, anterior-middle silk gland; MMSG, middle-middle silk gland; PMSG, posterior middle silk gland; PSG, posterior silk gland; Br, brain.

Due to the lack of annotated features, it is still challenging to predict putative function of lncRNAs merely from their sequence features. Fortunately, co-expression network-based "guilt-by-association" analysis methods have been successfully applied to the prediction of lncRNA function [85]. Therefore, we used WGCNA [62], an R package for weighted correlation network analysis, to associate lncRNAs with functional annotated mRNAs and predict their functions using a module-based method. The analysis resulted in 19 distinct modules with module sizes ranging from 17 to 4,248 (mean, 732; median, 123). Intriguingly, 12 of these 19 modules were strongly associated with tissue type (correlation > 0.65, *p*-value < = 0.03, Fig 6A, S5 and S6 Figs). Functional annotation and enrichment revealed that tissue-associated modules are biologically meaningful and related to tissue-specific biological processes (Fig 6, S7 Table).

The largest module (the blue module), which was specifically associated with the embryo (tissue correlation = 0.94, *p*-value = 2e-10), contained 1,586 lncRNAs and 2,662 mRNAs. In this module, genes related to “DNA binding” (GO:0003677), “DNA replication” (GO:0006260), “regulation of transcription, DNA-templated” (GO:0006355) were overrepresented and “Ubiquitin-mediated proteolysis” (bmor04120), “Spliceosome” (bmor03040), “Wnt signaling pathway” (bmor04310) and “FoxO signaling pathway” (bmor04068) were enriched, suggesting that lncRNAs play important roles in early embryonic development in the silkworm **(**S7 Table**)**.

In the aquamarine module (tissue correlation = 0.99, *p*-value = 8e-20), which was highly correlated with the brain, genes related to “hormone activity” (GO:0005179), “signal transduction” (GO:0007165), “neuropeptide signaling pathway” (GO:0007218), “synaptic transmission” (GO:0007268) and “Neuroactive ligand-receptor interaction” (bmor04080) were overrepresented, indicating that the functional enrichment results were consistent with brain attributes, and that the lncRNAs expressed in this module are involved in functional processes in the brain (S7 Table). The blueviolet module, which was enriched in “microtubule” (GO:0005874), “microtubule motor activity” (GO:0003777), “cilium morphogenesis” (GO:0060271), “microtubule-based movement” (GO:0007018), was highly correlated with testis (tissue correlation = 0.99, *p*-value = 3e-18), suggesting that lncRNAs in this module may play important roles in the spermatogenesis and the development of the testis (S7 Table). In addition, chartreuse, brown, cyan, chocolate, navy, and lightcoral, were specifically associated with the Malpighian tubules, integument, fatbody, ASG, ovary, and MSG, respectively (Fig 6, S5A Fig).

Notably, eight modules were related to the silk gland. Among these modules, the chocolate module was specific to ASG, and the dodgerblue module, lightcoral module, and orange module were highly correlated to AMSG, MMSG, and PMSG, respectively (S5B and S6 Figs). Given that the darkgoldenrod module is associated with both PMSG (tissue correlation = 0.49, *p*-value = 0.03) and PSG (tissue correlation = 0.65, *p*-value = 0.001), it is expected that the darkgoldenrod module is highly correlated with MPSG (tissue correlation = 0.84, *p*-value = 2e-06). The chocolate module, which is associated with ASG, contained 829 transcripts (470 mRNAs and 359 lncRNAs) and was found to be enriched in the “gluconeogenesis” (GO:0006094), “glycolytic process” (GO:0006096) biological processes, and the “Glycolysis/Gluconeogenesis”(bmor00010), and “Carbon metabolism” (bmor01200) pathways. Among these modules, darkgoldenrod module was mainly enriched in translation and protein export. Specifically, KEGG enrichment results showed the darkgoldenrod module enriched in “Ribosome”(bmor03010) and “Aminoacyl-tRNA biosynthesis”(bmor00970) pathways which were highly involved in translation process, and enriched in “Protein export”(bmor03060) and “Protein processing in endoplasmic reticulum”(bmor04141) pathways which represented protein export processing. In the dodgerblue module, which consists of 224 transcripts (118 mRNAs and 106 lncRNAs) and is specific to AMSG, genes involved in the “Folate biosynthesis” (bmor00790) pathway, “oxidation-reduction process” (GO:0055114), and “Mo-molybdopterin cofactor biosynthetic process”(GO:0006777) are overrepresented. The lightcoral module was enriched in the "Mucin type O-Glycan biosynthesis" (bmor00512), "Biosynthesis of unsaturated fatty acids" (bmor01040), and "Fatty acid metabolism" (bmor01212) pathways, suggesting that MSG not only participates in sericin biosynthesis but is also involved in the production of other components of the cocoon, e.g. fatty acids (S7 Table).

### Silkworm lncRNAs function as regulators of silk protein biosynthesis and secretion

As described above, eight modules was found to be associated with the silk gland. Since the darkgoldenrod module was the only module mainly enriched in translation and protein export, we highlighted this module to deeply investigate the functional role of lncRNAs in the silk gland. Based on the knowledge of coding gene annotation, Gene Ontology enrichment, KEGG pathway enrichment and cell biology, we manually split the darkgoldenrod gene network into translation-, translocation-, secretory-, cellular-, protein protection-, and unknown sub-function-related networks. All the sub-networks, except for the unknown sub-network, were selected for further analysis.

**Fig 7:**
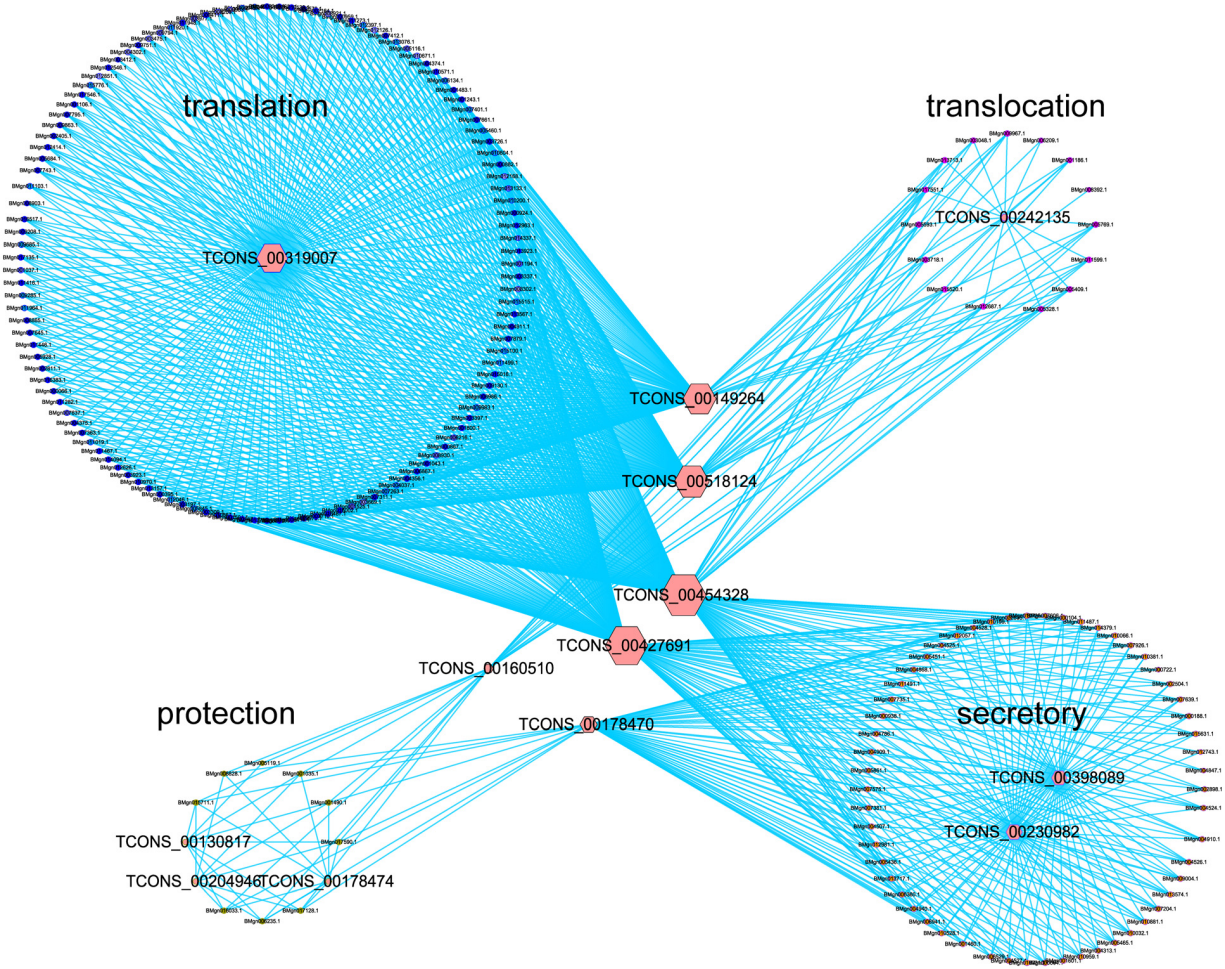
Network visualization of sub-networks derived from the darkgoldenrod module. Blue circular nodes represent protein-coding mRNAs and red hexagon nodes represent lncRNA hub genes. The network was grouped into four sub-networks (translation, translocation, secretory, and protection), displayed as four large circles. The node size represents the degree of connectivity of a particular gene. This image was created using Cytoscape software [63].

The translation sub-network was the largest, consisted of 128 coding genes, which were mainly involved in ribosome biogenesis, translation, formation of the translation pre-initiation complex, translation initiation, translational elongation, and aminoacyl-tRNA ligase. The secretory network, which was the second largest sub-network, consisted of 64 coding genes that were mainly involved in protein export, endoplasmic reticulum organization, endoplasmic reticulum unfolded protein response, and transmembrane transport. The translocation sub-network, consisting of the signal recognition particle (SRP), signal peptidase complex, translocon, and translocon-associated proteins, was ranked as the 3^rd^ sub-network. The fourth sub-network, namely the cellular and protein protection network, was composed of three negative regulators of macroautophagy proteins and seven serine protease inhibitors (Fig 7, S8 Table).

For each sub-network, the first neighboring lncRNAs of the well-annotated proteins were selected, and the top 5 lncRNAs in degree values from each four sub-networks were defined as hub genes, leading to the identification of 13 hub lncRNAs (7 intra-network hub lncRNAs and 6 inter-network hub lncRNAs) (Fig 7, S8 Table). Six of the hub lncRNAs were involved in at least two sub-networks. TCONS_00454328, with the highest degree, was in the module network core and participated in translation, translocation and secretion-related processes. TCONS_00149264 and TCONS_00518124 function as hub genes of the translation and translocation sub-network. TCONS_00427691 functions as a hub gene of the translation and secretory sub-network. In addition, seven lncRNAs were specific to each sub-network. For example, TCONS_00319007, with degrees of 122, was specific to the translation sub-network.

In addition to sub-network analysis, 17 lncRNAs of the darkgoldenrod module were found in the GROSS (genomic regions of selective signals) regions, and 10 lncRNAs were differentially expressed between domestic and wild silkworm strains. However, all of the above lncRNAs did not overlap with the 13 selected hub genes, suggesting that the 13 selected hub genes play baseline functional roles in silk protein synthesis and secretion. Additionally, several genes involved in the juvenile hormone (JH) pathway were identified. More recently, our group demonstrated that JH is involved in silk protein synthesis [86], suggesting that lncRNAs may interact with the JH pathway and participate in regulating silk protein synthesis.

Silk proteins are mainly synthesized in MSG and PSG. In 48~96-hour-old fifth instar larvae, an increase in the numbers of rough ER and Golgi vacuoles were observed [87]. Based on comparative proteomic analysis, the Zhong group showed that the aminoacyl-tRNA biosynthesis, ribosome, and secretory pathways are significantly enriched in MSG and PSG at the third day of the fifth instar larval stage [88]. Previously, using SAGE-aided transcriptomic analysis, the Pierre group found highly abundant transcripts, in both MSG and PSG cells, which encoded ribosomal proteins and translation factors [89]. Moreover, small RNA-seq of PSG showed that some miRNAs may be involved in the synthesis of silk protein [33]. Collectively, the 13 hub lncRNAs, especially the six inter-network hub lncRNAs, may function as regulators of silk protein biosynthesis and secretion.

## Conclusion

In the present study, we identified 11,810 lncRNAs in the silkworm, including 6, 250 lincRNAs, 474 ilncRNAs, and 5,086 lncNATs, by integrative analysis of 21 relatively high-depth and high-quality RNA-seq libraries. The genomic features of the identified lncRNAs were examined. We found that silkworm lncRNAs were shorter in terms of overall length, with longer exons and introns, smaller exon pre-transcripts, harbored more transposons, higher SNP density, and relatively low levels of expression compared with silkworm protein-coding mRNAs. Several limitations were existed in the present study. As most of the RNA-seq libraries generated were non strand-specific and poly(A)-selected, a large proportion of non-poly(A) silkworm lncRNAs were not detected. In addition, single-exon transcripts were excluded due to lack of availability of strand information. The number of ilncRNAs and lncNATs may have been underestimated, and the transcript lengths and exon numbers per transcript of lncRNAs overestimated. Additionally, some bona fide transcripts may have been lost. In the current study, we analyzed sequence conservation rather than positional conservation. Therefore, further investigation based on phylogenomics approaches are warranted for elucidation of the functional roles of conserved lncRNAs.

Like lncRNAs in other species, silkworm lncRNAs tend to be expressed in a tissue-specific manner, and may function as miRNA precursors or ceRNAs. Sexual dimorphism was also investigated, and 49.47% of lncRNA loci (2,749) were found to be expressed in a sex-biased manner. Co-expression network analysis showed that 12 out of 19 modules exhibited relatively high association with specific tissue types. Moreover, functional enrichment results suggested that the tissue-associated modules are biologically meaningful and related to tissue-specific biological processes. In-depth analysis of the highlighted darkgoldenrod module, which is specifically associated with the middle and posterior silk gland where main places of silk protein biosynthesis is, suggested that the hub lncRNAs of this module may function as regulators of silk protein biosynthesis, translocation, and secretion. This study presents the first comprehensive genome-wide analysis of silkworm lncRNAs and provides an invaluable resource for genetic, evolutionary, and genomic studies of the silkworm. Moreover, our findings are expected to provide new insights into the mechanisms underlying the biosynthesis of silk protein.

## Supporting Information

S1 Fig Genomic characterization of silkworm lncRNAs. (A) Distribution of lncRNAs along each chromosome: (a) percentage of repetitive sequences in 200-kb windows; (b) number of miRNAs in 200-kb windows; (c) number of protein-coding mRNAs in 200-kb windows; (d) number of lincRNAs in 200-kb windows; (e) number of lncNATs in 200-kb windows; (f) number of ilncRNAs in 200-kb windows. (B) Seqlogo of nucleotide frequencies at donor and acceptor sites of lncRNAs and protein-coding mRNAs. (C) Conservation of silkworm lncRNAs and protein-coding mRNAs. (a) The heatmap presents the homolog sequence fragment identified across 17 other selected genomes for lncRNAs. (b) The heatmap presents the homolog sequence fragment identified across 17 other selected genomes for protein-coding mRNAs. (c) The number of homolog sequences discovered for each lncRNA and protein-coding mRNAs. (D) The cumulative density of lncNAT sequences overlapped by mRNAs. (E) SNP density of different types of transcripts.
(PDF)

S2 Fig Characteristics of lncRNA expression in silkworm tissues. (A) Distribution of expression log10 (FPKM+1) of lincRNA (red), lncNAT (blue) and protein-coding (green) mRNAs in 21 silkworm tissues. (B) Density distribution of maximum expression levels for lincRNAs, lncNATs, and protein-coding mRNAs in 21 different silkworm tissues.
(PDF)

S3 Fig LncRNAs as potential competing endogenous RNAs. (A) Schematic of conservation elements region of XLOC_004695 gene locus that harbor bmo-miR-184 response elements. The purple box shows the region contains conservation sequence elements. The consensus logo highlights the 240-bp conserved sequence, which was identified from the 8 insect genome alignments. The sequences alignments represent for bmo-miR-184 response elements, the above vertical lines indicating Watson—Crick base pairs. (B) The expression pattern of potential competing endogenous RNA (TCONS_00111202) in the 21 silkworm tissues.
(PDF)

S4 Fig Sex-biased alternatively spliced variant expression of lncRNA locus XLOC_012091 in silkworm. (A) Gene structure of XLOC_012091 locus and its antisense overlap protein-coding gene BMgn007254.1. (B) Expression pattern of transcript isoforms of the XLOC_012091 gene locus.
(PDF)

S5 Fig WGCNA modules and relationship between module eigengenes (MEs) and the traits. (A) Relationships between module eigengenes (MEs) and traits. Horizontally, MEs are named according to module color. Vertically, traits of interest are listed (Sex, Female, Male, segments of silk gland (ASG, AMSG, MMSG, PMSG, and PSG), combination of adjacent silk gland parts, and other type of tissues). The correlation coefficients between the respective ME and the trait of interest, and the corresponding *p*-values (in parentheses), are shown in the boxes. The deeper the red color of the box, the more positive the correlation with the trait. Inversely, a deeper shade of green indicates a more negative correlation with the trait. (B) Relationships between silk gland-specific module eigengenes (MEs) and traits. Traits of interest are listed (Female, Male, combination of adjacent silk gland parts). MpPSG, PMSG+PSG; MmpPSG, (MMSG+PMSG+PSG); MmpSG, (MMSG+PMSG); AMaSG, (ASG_AMSG).
(PDF)

S6 Fig Expression pattern of all genes in 12 selected modules across all 21 tissues. Heatmap in the upper panel showing the expression pattern of all genes in this module across all 21 tissues. Red, representing increased expression; black, representing neutral expression; green, representing decreased expression. Barplot in the middle panel showing the values of the module eigengene versus each tissues. Pie charts in the bottom panel indicating the number of mRNAs and lncRNAs within this module.
(PDF)

S1 Table RNA-seq datasets. (XLSX)

S2 Table Genomic information for the identified silkworm lncRNAs (GTF format). (XLSX)

S3 Table The chromosome distribution of silkworm lncRNAs. (XLSX)

S4 Table Summary of lncRNAs function as putative miRNA precursors. (XLSX)

S5 Table Summary of lncRNAs with putative miRNA response regions. (XLSX)

S6 Table Summary of sex-biased expression of lncRNA and mRNA. (XLSX)

S7 Table Module members and functional enrichment of protein-coding genes in each modules. (XLSX)

S8 Table Sub-networks and gene annotation of darkgoldenrod module is shown in Fig 7. (XLSX)
