# Supplementary figures and images for: Systematic Identification and Characterization of Long Non-Coding RNAs in the Silkworm, Bombyx mori
Source: PLoS One. 2016 Jan 15;11(1):e0147147. doi: 10.1371/journal.pone.0147147 (PMC4714849; doi:10.1371/journal.pone.0147147)

**A**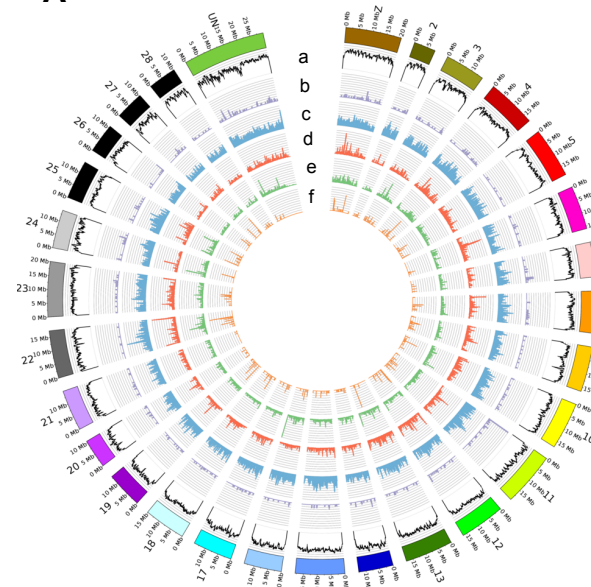**B**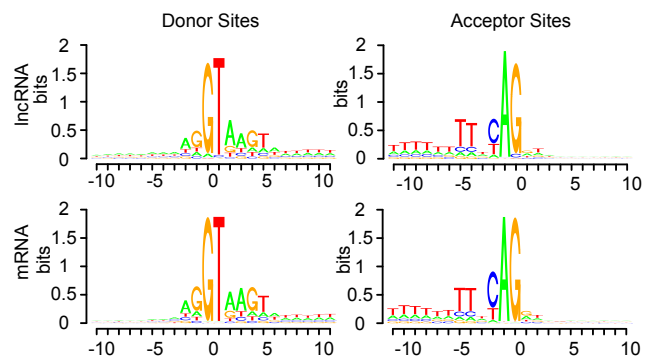**C**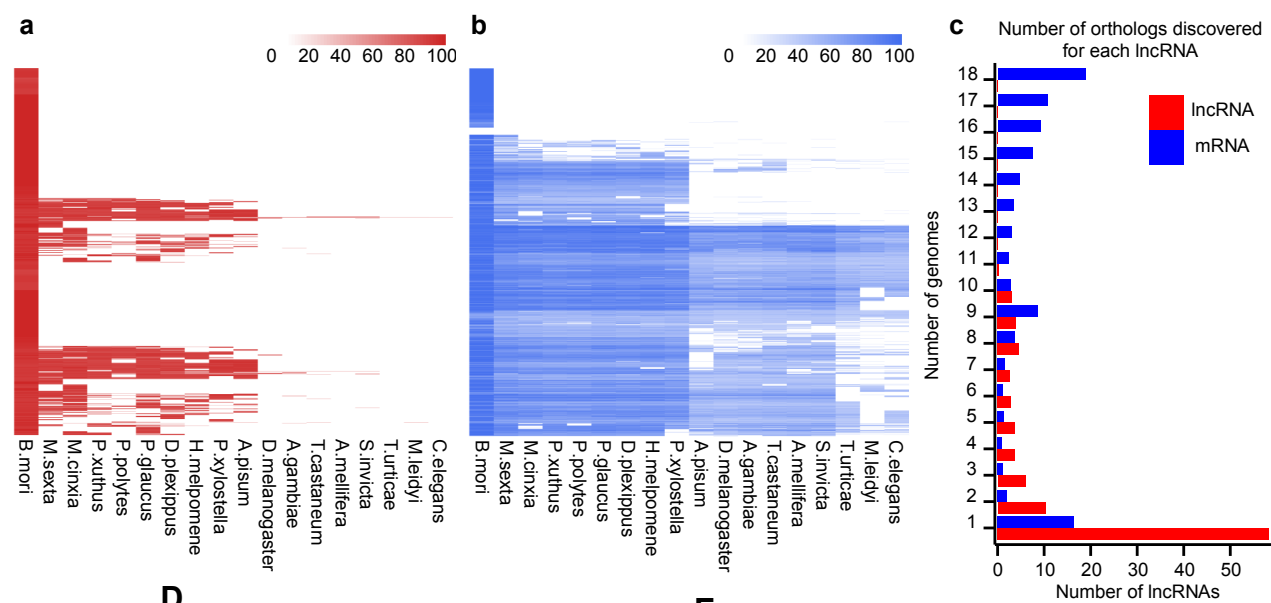**D**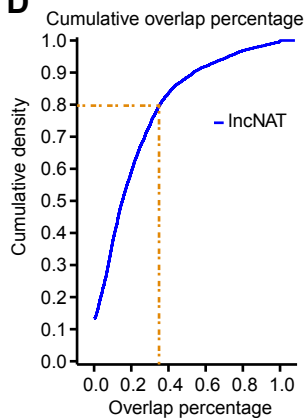**E**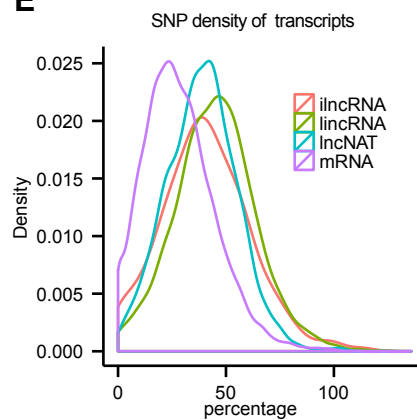

Supplement: S1 Fig — (A) Distribution of lncRNAs along each chromosome: (a) percentage of repetitive sequences in 200-kb windows; (b) number of miRNAs in 200-kb windows; (c) number of protein-coding mRNAs in 200-kb windows; (d) number of lincRNAs in 200-kb windows; (e) number of lncNATs in 200-kb windows; (f) number of ilncRNAs in 200-kb windows. (B) Seqlogo of nucleotide frequencies at donor and acceptor sites of lncRNAs and protein-coding mRNAs. (C) Conservation of silkworm lncRNAs and protein-coding mRNAs. (a) The heatmap presents the homolog sequence fragment identified across 17 other selected genomes for lncRNAs. (b) The heatmap presents the homolog sequence fragment identified across 17 other selected genomes for protein-coding mRNAs. (c) The number of homolog sequences discovered for each lncRNA and protein-coding mRNAs. (D) The cumulative density of lncNAT sequences overlapped by mRNAs. (E) SNP density of different types of transcripts. (PDF) [file pone.0147147.s001.pdf]

**A**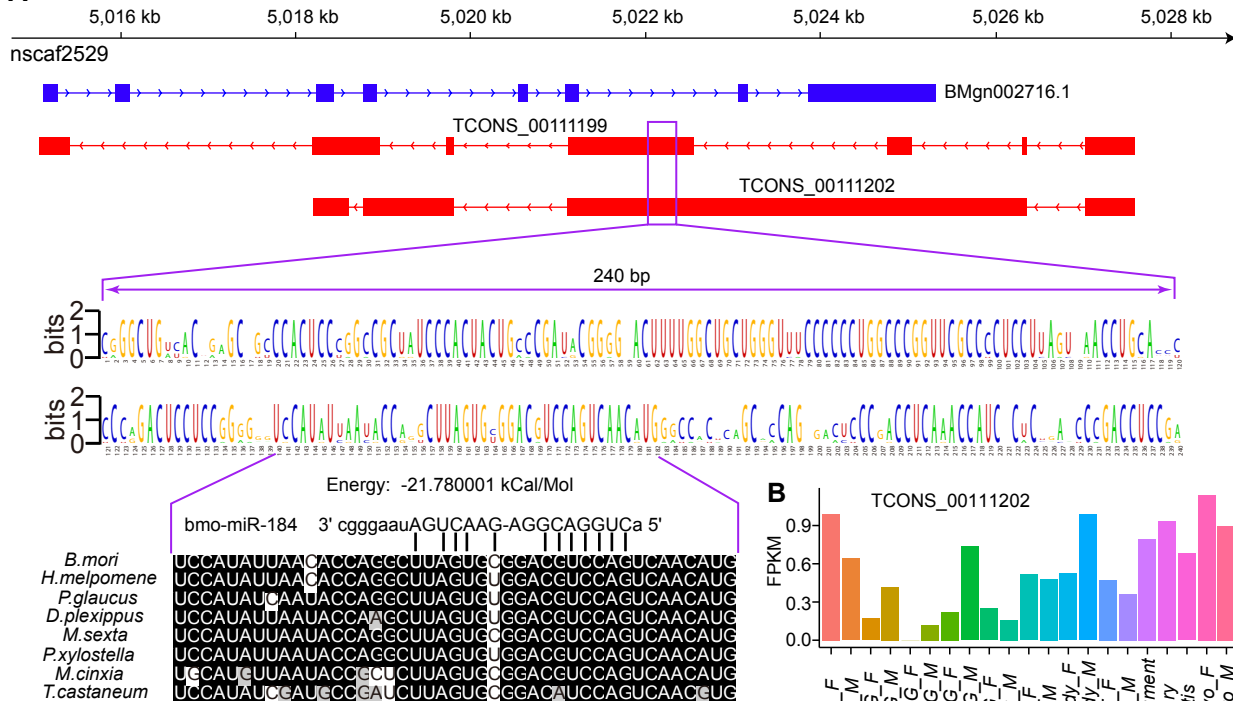**B**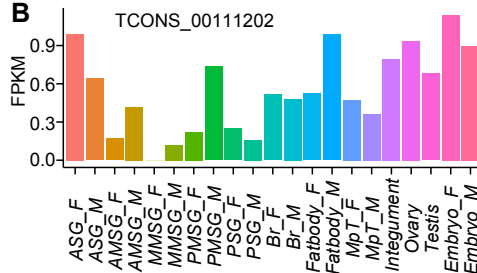

Supplement: S3 Fig — (A) Schematic of conservation elements region of XLOC_004695 gene locus that harbor bmo-miR-184 response elements. The purple box shows the region contains conservation sequence elements. The consensus logo highlights the 240-bp conserved sequence, which was identified from the 8 insect genome alignments. The sequences alignments represent for bmo-miR-184 response elements, the above vertical lines indicating Watson—Crick base pairs. (B) The expression pattern of potential competing endogenous RNA (TCONS_00111202) in the 21 silkworm tissues. (PDF) [file pone.0147147.s003.pdf]

nscf2876

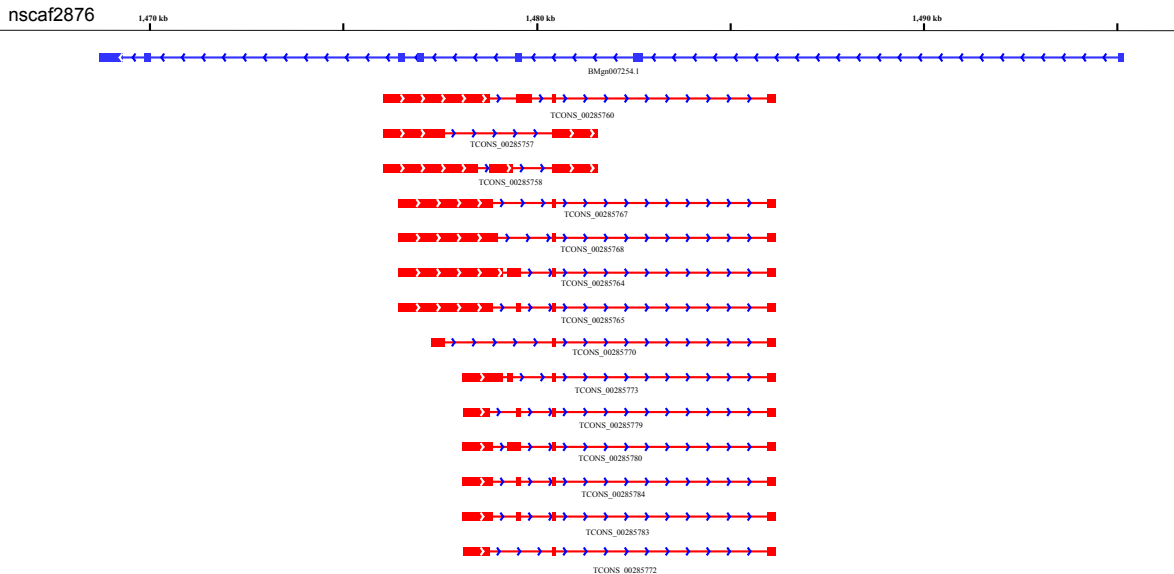[illegible]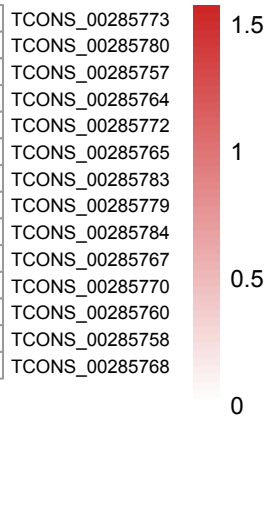

Supplement: S4 Fig — (A) Gene structure of XLOC_012091 locus and its antisense overlap protein-coding gene BMgn007254.1. (B) Expression pattern of transcript isoforms of the XLOC_012091 gene locus. (PDF) [file pone.0147147.s004.pdf]

**A**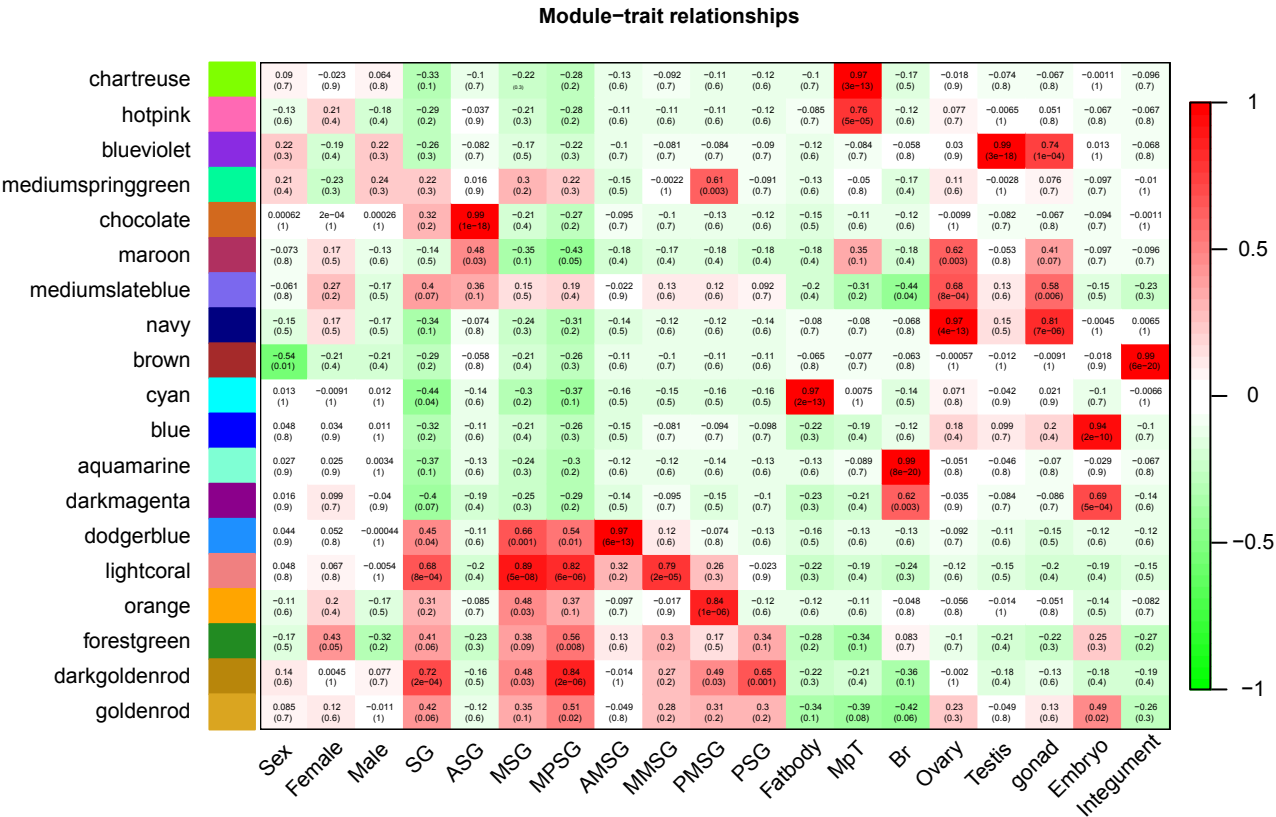**B**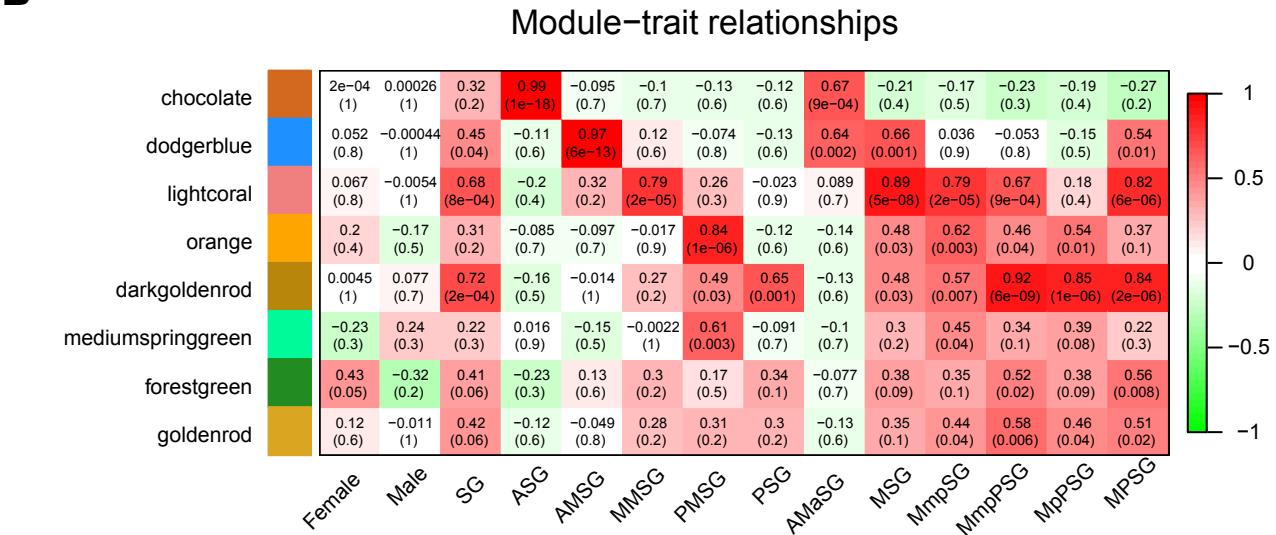

Supplement: S5 Fig — (A) Relationships between module eigengenes (MEs) and traits. Horizontally, MEs are named according to module color. Vertically, traits of interest are listed (Sex, Female, Male, segments of silk gland (ASG, AMSG, MMSG, PMSG, and PSG), combination of adjacent silk gland parts, and other type of tissues). The correlation coefficients between the respective ME and the trait of interest, and the corresponding p-values (in parentheses), are shown in the boxes. The deeper the red color of the box, the more positive the correlation with the trait. Inversely, a deeper shade of green indicates a more negative correlation with the trait. (B) Relationships between silk gland-specific module eigengenes (MEs) and traits. Traits of interest are listed (Female, Male, combination of adjacent silk gland parts). MpPSG, PMSG+PSG; MmpPSG, (MMSG+PMSG+PSG); MmpSG, (MMSG+PMSG); AMaSG, (ASG_AMSG). (PDF) [file pone.0147147.s005.pdf]
