# Supplementary material for: Systematic Identification and Characterization of Long Non-Coding RNAs in the Silkworm, Bombyx mori
Source: PLoS One. 2016 Jan 15;11(1):e0147147. doi: 10.1371/journal.pone.0147147 (PMC4714849; doi:10.1371/journal.pone.0147147)
Supplement: S2 Fig — (A) Distribution of expression log10 (FPKM+1) of lincRNA (red), lncNAT (blue) and protein-coding (green) mRNAs in 21 silkworm tissues. (B) Density distribution of maximum expression levels for lincRNAs, lncNATs, and protein-coding mRNAs in 21 different silkworm tissues. (PDF) [file pone.0147147.s002.pdf]

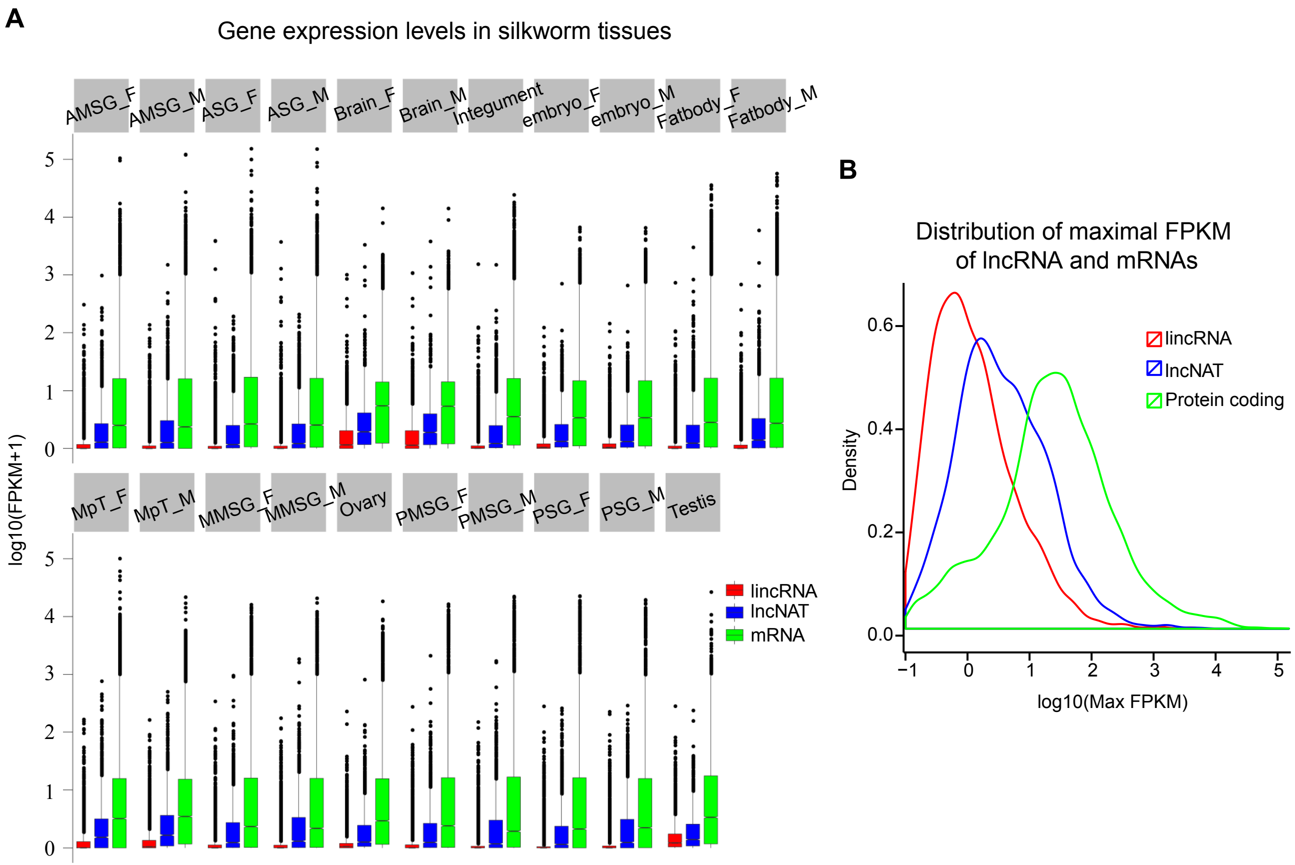
A
Gene expression levels in silkworm tissues
AMSG_F
AMSG_M
ASG_F
ASG_M
Brain_F
Brain_M
Integument
embryo_F
embryo_M
Fatbody_F
Fatbody_M
MpT_F
MpT_M
MMSG_F
MMSG_M
Ovary
PMSG_F
PMSG_M
PSG_F
PSG_M
Testis
log10(FPKM+1)
lincRNA
lncNAT
mRNA
B
Distribution of maximal FPKM of lncRNA and mRNAs
Density
lincRNA
lncNAT
Protein coding
log10(Max FPKM)
