# Supplementary material for: Systematic Identification and Characterization of Long Non-Coding RNAs in the Silkworm, Bombyx mori
Source: PLoS One. 2016 Jan 15;11(1):e0147147. doi: 10.1371/journal.pone.0147147 (PMC4714849; doi:10.1371/journal.pone.0147147)
Supplement: S6 Fig — Heatmap in the upper panel showing the expression pattern of all genes in this module across all 21 tissues. Red, representing increased expression; black, representing neutral expression; green, representing decreased expression. Barplot in the middle panel showing the values of the module eigengene versus each tissues. Pie charts in the bottom panel indicating the number of mRNAs and lncRNAs within this module. (PDF) [file pone.0147147.s006.pdf]

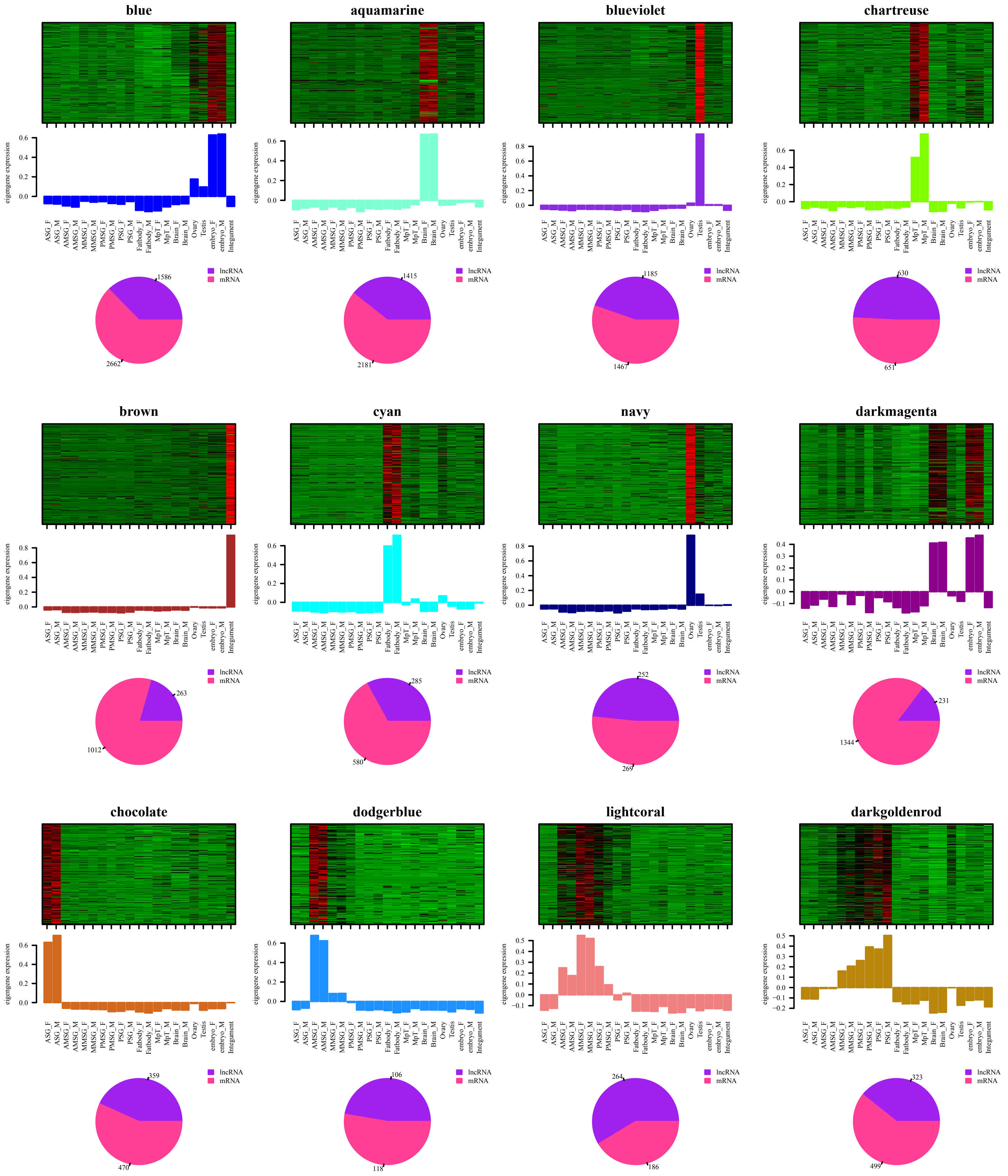
blue
eigengene expression
0.0
0.2
0.4
0.6
ASG_F
ASG_M
AMSG_F
AMSG_M
MMSG_F
MMSG_M
PMSG_F
PMSG_M
PSG_F
PSG_M
Fatbody_F
Fatbody_M
MpT_F
MpT_M
Brain_F
Brain_M
Ovary
Testis
embryo_F
embryo_M
Integument
lncRNA
mRNA
1586
2662
aquamarine
1415
2181
blueviolet
0.8
1185
1467
chartreuse
630
651
brown
263
1012
cyan
285
580
navy
252
269
darkmagenta
0.1
0.3
-0.1
231
1344
chocolate
359
470
dodgerblue
106
118
lightcoral
0.5
264
186
darkgoldenrod
-0.2
323
499
